# Pressure-induced p–n conductivity type switching and self-driven photocurrent polarity reversal in BiI_3_

**DOI:** 10.1093/nsr/nwaf192

**Published:** 2025-05-29

**Authors:** Zexiang Shen

**Affiliations:** School of Physical & Mathematical Sciences, Nanyang Technological University, Singapore

Diodes are typically realized by combining two semiconductors with different conduction types, or at least by defining different doping levels within the device [1,2]. As the miniaturization of diodes and transistors becomes increasingly challenging, alternative design strategies are urgently needed. One promising approach is to achieve reversible conductivity transitions within a single material system [3]. Recently, a novel class of inorganic semiconductors has been identified that exhibits remarkable switching between p-type and n-type conduction in response to temperature variations, offering significant potential for transistor and memory applications [4]. Pressure, similar to temperature, is a key thermodynamic parameter capable of continuously modulating a material's crystal and electronic structures. Therefore, pressure-driven p–n switching should also be feasible. However, most semiconductors tend to undergo metallization under high pressure, which severely limits their practical applications. Consequently, it is critical to identify materials that can undergo rapid carrier-type transitions under pressure via semiconductor-to-semiconductor phase transformations.

**Figure 1.**
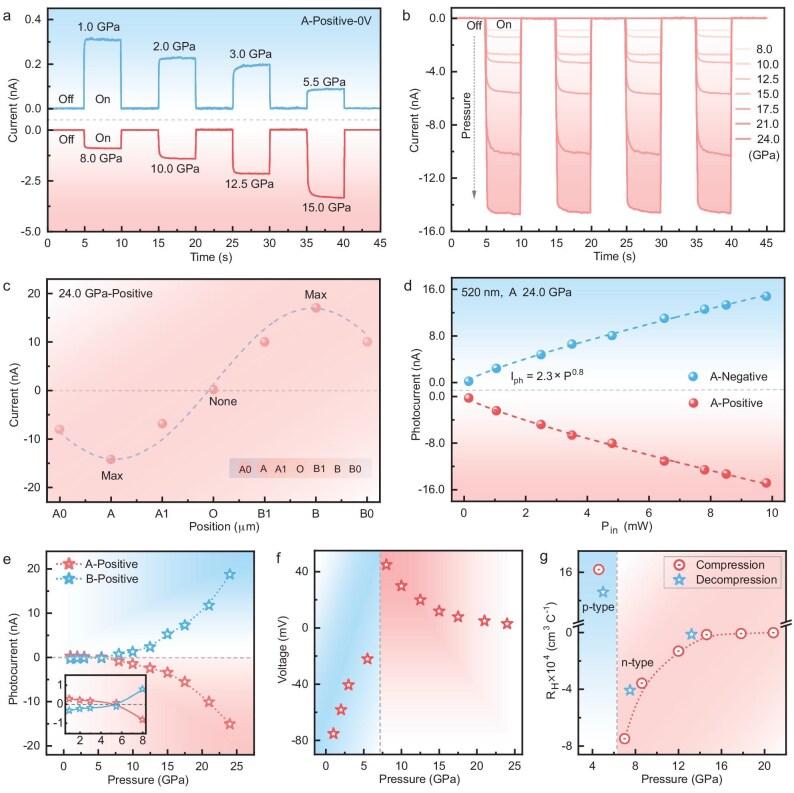
(a and b) Photoresponse of BiI_3_ under localized laser illumination at position A with zero bias under pressure. (c) Spatial variation of the photocurrent at 24.0 GPa. (d) Laser intensity dependence of the photocurrent at 24.0 GPa. (e) Overall pressure dependence of the photocurrent. (f) Evolution of the photothermoelectric voltage with pressure. (g) Pressure-dependent variation of the Hall coefficient of BiI_3_. Reproduced with permission from Ref. [5].

Liu *et al*. reported dramatic, reversible p–n switching in semiconducting BiI_3_ via pressure modulation (Fig. 1a and b) [5]. Notably, even after the transition, BiI_3_ retained a suitable bandgap of 1.14 eV—a significant departure from previously studied semiconductors, which often experienced bandgap closure during such transitions. Additionally, the authors proposed that the carrier type under high pressure can be determined by photocurrent measurements dominated by the photothermoelectric (PTE) effect (Fig. 1c and d). The polarity of this photocurrent, induced by spontaneous carrier movement due to concentration gradients under non-uniform illumination, excludes any influence from an applied bias voltage. The unique characteristics of BiI_3_, including rapid carrier switching and the retention of a suitable bandgap (Fig. 1e–g), provide an unprecedented opportunity for optoelectronic device applications. In the future, artificially designed pressure gradients within the diamond anvil cell (DAC) are expected to construct single-component p–n junctions. In addition, reversal of the polarity of the optical signal of BiI_3_ under pressure also enables the design of pressure-responsive switches.

In summary, Liu and co-workers highlighted the effective and flexible modulation of carrier properties through pressure engineering. Their study introduced a novel method for identifying carrier polarity via photoelectric measurements and revealed the relationship between positive/negative photoconductance switching and carrier type under high pressure. This study not only deepened the understanding of carrier behavior in semiconductors, but also provided valuable insights into the design of logic circuits and the optimization of device performance.
